# Oct-2 forms a complex with Oct-1 on the iNOS promoter and represses transcription by interfering with recruitment of RNA PolII by Oct-1

**DOI:** 10.1093/nar/gkv829

**Published:** 2015-08-13

**Authors:** Fatima Bentrari, Aurelie Chantôme, Andrew Knights, Jean-François Jeannin, Alena Pance

**Affiliations:** 1 EPHE Laboratory, Faculty of Medicine, University of Bourgogne, 7 Boulevard Jeanne D'Arc, 21033 Dijon, France; 2 The Wellcome Trust Sanger Institute, Genome Campus, Hinxton, Cambridge CB10 1SA, UK

## Abstract

Oct-1 (POU2f1) and Oct-2 (POU2f2) are members of the POU family of transcription factors. They recognize the same DNA sequence but fulfil distinct functions: Oct-1 is ubiquitous and regulates a variety of genes while Oct-2 is restricted to B-cells and neurones. Here we examine the interplay and regulatory mechanisms of these factors to control the inducible nitric oxide synthase (iNOS, NOS2). Using two breast cancer cell lines as a comparative model, we found that MCF-7 express iNOS upon cytokine stimulation while MDA-MB-231 do not. Oct-1 is present in both cell lines but MDA-MB-231also express high levels of Oct-2. Manipulation of Oct-2 expression in these cell lines demonstrates that it is directly responsible for the repression of iNOS in MDA-MB-231. In MCF-7 cells Oct-1 binds the iNOS promoter, recruits RNA PolII and triggers initiation of transcription. In MDA-MB-231 cells, both Oct-1 and Oct-2 bind the iNOS promoter, forming a higher-order complex which fails to recruit RNA PolII, and as a consequence iNOS transcription does not proceed. Unravelling the mechanisms of transcription factor activity is paramount to the understanding of gene expression patterns that determine cell behaviour.

## INTRODUCTION

One of the fundamental aspects of gene expression is the understanding of how the various mechanisms of transcriptional regulation control specific genes in a tissue-specific manner. Whether transcription is controlled at the step of pre-initiation complex (PIC) formation and RNA Polymerase II (PolII) recruitment, initiation, elongation or termination varies from gene to gene and is largely determined by the combination of transcription factors present in a cell in particular circumstances. Thus there are general transcription factors that form part of the basal machinery, ubiquitous transcription factors that are present in many tissues and others whose expression is restricted to specific cell types. The combinatorial binding and interaction of the transcription factors expressed in a cell at a particular time integrates extracellular signals and orchestrates the cell response.

The Pit-Oct-Unc (POU) family of transcription factors includes many proteins that have been grouped in seven distinct classes according to function and expression containing proteins such as the neuronally expressed Pit proteins in class I, the ubiquitously expressed Oct-1 and tissue-specific Oct-2 in the second category, the well-known pluripotency marker Oct-4 which belongs to class V, etc; (1). The family is characterized by a central DNA-binding domain which in many members has affinity for the same octamer element ATGCAAAT. The POU DNA-binding domain is flanked by distinct activation domains characteristic of each member (2). The bipartite DNA-binding domain consists of an N-terminus sub-domain of the protein (POU specific domain) that contacts the ATGC sequence of the Oct element, while the C-terminus or POU homeodomain rests in the AAAT site major groove. The two domains are tethered by a flexible linker that varies between the family members and gives intrinsic conformational flexibility of binding to the consensus octamer DNA element and variants of it. With the two domains and their flexible linker, the protein envelops the DNA and the sub-domains are able to adapt their conformations to the DNA element and to the presence of other proteins.

Oct-1 (POU2f1) is a ubiquitous factor present in a wide range of cell types while Oct-2 (POU2f2) expression has been found confined to B lymphocytes and neuronal cells. The POU domain is 87% identical between Oct1 and Oct2 with the main differences being in the linker region and as a consequence they have identical DNA-binding specificity to the consensus element. To either side of the POU domain are the activation domains, of much less significant similarity between the two factors, although the N-terminal regions are both glutamine-rich and the C-termini have high content of serines threonines and prolines (3). Functionally, Oct-2 has been implicated in B cell development and regulation of B lymphocyte-specific genes. In agreement with its wide distribution, Oct-1 has been attributed a role in the response to stress and the regulation of house-keeping and similarly ubiquitous genes such as H2B (4). Both transcription factors are multifunctional molecules that can repress or activate gene expression depending on the context of the particular promoter and the cell type where they are expressed (5,6). As a result of this complexity, the mechanisms by which they regulate gene expression and how their activities complement each other remain unclear.

We showed previously that Oct-1 is essential for expression of the human inducible nitric oxide synthase (NOSII, iNOS) (7). It binds to the proximal promoter just upstream of the TATA box and an important part of its role is the assembly of the PIC leading to transcription initiation and PolII pausing. iNOS is ubiquitously expressed throughout numerous cell types only in response to immune stimuli. The high levels of nitric oxide (NO) produced by iNOS are part of the inflammatory response and innate immunity, being able to kill microorganisms and neoplastic cells in a non-specific manner (8). NO is a highly reactive signalling molecule that influences cell death and survival. Due to the toxicity of the high levels of the NO it produces, expression of iNOS is tightly regulated so recruitment of PolII and initiation of transcription ensure that the enzyme is produced rapidly and exclusively where needed. This is achieved by translating extracellular stimuli into activation of transcription factors such as NFkB, AP-1 and STAT-1, which then rapidly trigger elongation (9).

In this work, we investigate the observation that cell lines where Oct-2 is present lose the capacity to induce iNOS. We use two breast cancer cell lines as a comparative model: MCF-7 and MDA-MB-231. We show that MCF-7 cells behave similarly to the colon cancer line HCT-8R that we used in our previous work in that it expresses Oct-1, which binds constitutively to the proximal iNOS promoter to facilitate recruitment of PolII and initiation of transcription as revealed by transcription of exon1. In contrast, MDA-MB-231 cells express Oct-2 in addition to Oct-1 and iNOS is not responsive to cytokine stimulation. We confirmed the role of Oct-2 in iNOS repression by knockdown in MDA-MB-231 cells which causes a recovery of inducibility and transfection of full length (FL) Oct-2 into MCF-7 cells which results in iNOS repression. Examining the mechanism of this regulation we found that both factors form a higher order complex on the iNOS promoter and as a consequence of the presence of Oct-2 PolII is not recruited and transcription of iNOS is blocked.

## MATERIALS AND METHODS

### Cell culture

All cell lines were maintained in Dulbecco's modified Eagle's medium supplemented with 10% foetal calf serum and 2% glutamine. A mix of recombinant human cytokines (CM): IL-1β (3 ng/ml) (Sanofi, France), IFN-γ (200 U/ml) (Roussel-Uclaf, France) and TNF-α (75 ng/ml) (Dainippon, Japan) were added to the same medium for the specified time.

### Plasmids

A pXP2 vector containing 7 kb of the iNOS promoter coupled to luciferase as a reporter gene was used to assess transcriptional activity (10). A pbluescript plasmid containing human hepatocyte iNOS cDNA was used for northern blotting. NFκB transcriptional activity was determined using a pNFκB-luciferase reporter containing four copies of the κB site in a pUC vector (11). The FL Oct-2 expression vector (pOEV1) was used for transfection (12).

### Cell transfection and reporter gene assay

Cells were plated on 6-well plates at 1 × 106 cells per cm^2^ to achieve 80% confluence. Fugene6 (Qiagen, UK) was used for transfection and reporter gene transcription was determined using the dual luciferase assay from Promega (Madison, USA) as described previously (13).

### RNA extraction northern blot and RT-PCR

A 600-bp fragment of the human NOSII cDNA (46–644) was used as a probe for iNOS detection by northern blot as described previously (7).

Total RNA from all samples was extracted with the Isolate II RNA mini kit (Bioline) following the instructions and 1–3 μg were reverse transcribed with a MuLV reverse transcriptase (Applied Biosystems, UK) using random primers (Bioline, UK). One microlitre of cDNA was specifically and quantitatively amplified using SybrGreen polymerase chain reaction (PCR) master mix (Applied Biosystmes, UK) following the cycling parameters established by the manufacturer on a light cycler 480 II (Roche) and using GAPDH as a control for normalization. The primers used (SIGMA) were (5′–3′):

**Table:** 

GAPDH:	F: gcctcctgcaccaccaactgc	R: ggcagtgatggcatggactg	102 bp
Exon 1:	F: gcagagaactcagcctcattc	R: ggtaaggacagtcaaaccag	126 bp
Exon 12:	F: gtgaccatcatggaccaccactcgg	R: ccaagactttcaatggaatc	265 bp
Oct-1:	F: acaggctgctgctcagtctt	R: ctgtcctccagctagcataagc	135 bp
Oct-2:	F: atggttcactccagcatgg	R: ggttctgatgattagtgtctgg	133 bp

### Electrophoretic mobility shift assays (EMSA)

The assays were performed as described previously (7,14). The oligonucleotide corresponding to the Octamer element of the proximal iNOS promoter in its sense and anti-sense orientation were hybridized and labelled with T4 polynucleotide kinase following manufacturer's instructions and purified on sephadex 50 columns (Boehringer Mannheim). The consensus elements for AP-1, Oct-1 as well as NFkB were obtained from Promega (Madison, WI, USA). The specific antibodies to the AP-1 family, Oct and NFkB were purchased from Santa Cruz Biotechnology Inc (CA, USA).

### Western blot

Cells were washed in phosphate buffered saline (PBS) and resuspended in sample buffer (Tris/HCl 0.5 M pH 6.8, 30% glycerol, 10% sodium dodecyl sulphate (SDS)), vortexed and centrifuged eliminating the cell debris. The total protein concentration was determined by the Lowry assay. A sodium dodecyl sulphate-polyacrylamide gel electrophoresis was performed, separating 30 μg of total in a 7% acrylamide/bis-acrylamide (30:1) gel at 80 V. The separated proteins were then transferred onto Polyvinylidene_fluoride (PVDF) membranes for 2 h at 250 mA and ∼4–5V.

The membranes were blocked in PBS containing 0.1% tween 20 and 5% skimmed milk for 1 h at room temperature. Then the first antibody was added in PBS containing 0.1% Tween 20 (PBS-T) incubating for 2 h at room temperature with gentle shaking. Three washes were performed with PBS-T 10 min each, and then the secondary antibody was added in PBS-T, incubating for 1 h. The reactions were developed with the horse radish peroxidase luminescence visualization system (ECL).

### NO biosynthesis determination

Cells were plated in 6-well plates in a proportion of 5 × 10E3/well in complete medium. They were depleted of hormones for 24–48 h and stimulated for 24 and 48 h. The supernatants were recovered and the concentration of NO_2_^−^ was determined by the Griess reaction (as described in 13). The NO3- were reduced to NO_2_^−^ by the nitrate reductase and these were measured using the same system.

### Vector construction

The pcDNA™3.1/V5-His TOPO^®^ TA Expression vector (Invitrogen, Paisley, UK) was used to clone amplified RT-PCR products. The primers used for the FL Oct-2 cDNA are (5′–3′):

**Table:** 

F: atggttcactccagcatgggg	R: aggctggtaaggggcaggg

And for the anti-sense Oct-2 fragment (5′ – 3′):

**Table:** 

F: tgttgacgggcagccagc	R: atgggaatagatttggtgtc

### Chromatin immunoprecipitation (ChIP)

Twenty million cells were lysed using a low SDS chromatin shearing kit (Diagenode), according to the manufacturer's instructions. Chromatin was sheared using a Bioruptor Plus (Diagenode) to obtain fragments ranging from 100 to 500 bp. Chromatin shearing was assessed using a 2100 Bioanalyzer (Agilent) with a high sensitivity chip, according to the manufacturer's instructions. Immunoprecipitation was performed as described previously (7) with the following antibodies:

anti-IgG (Rabbit control IgG-ChIP grade ab46540, anti-RNA PolII CTD repeat YSPTSPS (phospho S5) antibody—chromatin immunoprecipitation (ChIP) Grade (ab5131) and anti-RNA PolII CTD repeat YSPTSPS (phospho S2) antibody—ChIP Grade (ab5095) (Abcam) and anti-Oct1 sc-232X and anti-Oct2 sc-233 (Santa Cruz Biotechnology)). One microlitre of precipitated chromatin was specifically and quantitatively amplified using the SybrGreen system (Applied Biosystmes, UK) following the cycling parameters established by the manufacturer.

The primers for the qPCR analysis (as above) of binding to the iNOS promoter were (5′–3′):

**Table:** 

P2 F: AAGGCACAGGTCTCTTCCTGGTTT	R: AATGAAGGCAACTCACCTTGCAGC

And for the downstream iNOS gene (5′-3′):

**Table:** 

Pg F: TCTGTAGGAAGTGGGCAGGAGAAT	R: TGCTGTGCTCCATAGTTTCCAGGT

The Sequential ChIP was done as above but the precipitated chromatin was eluted with TE (75 μl) containing 1% SDS, fresh DTT (15 mM) and cOmplete protease inhibitors (Roche) for 30′ at 37°C. The beads were washed with dilution buffer (75 μl) and this eluate was pooled with the first. The total eluate was diluted 10× with dilution buffer, incubated at 4°C for 5 h and subjected to a second round of ChIP as described above.

### Statistical analysis

Values are presented as means and standard deviation. Any reported significance was calculated using *t*-test analysis and the indicated *P*-value.

## RESULTS

### iNOS is differentially expressed in breast cancer cell lines

**Figure 1.**
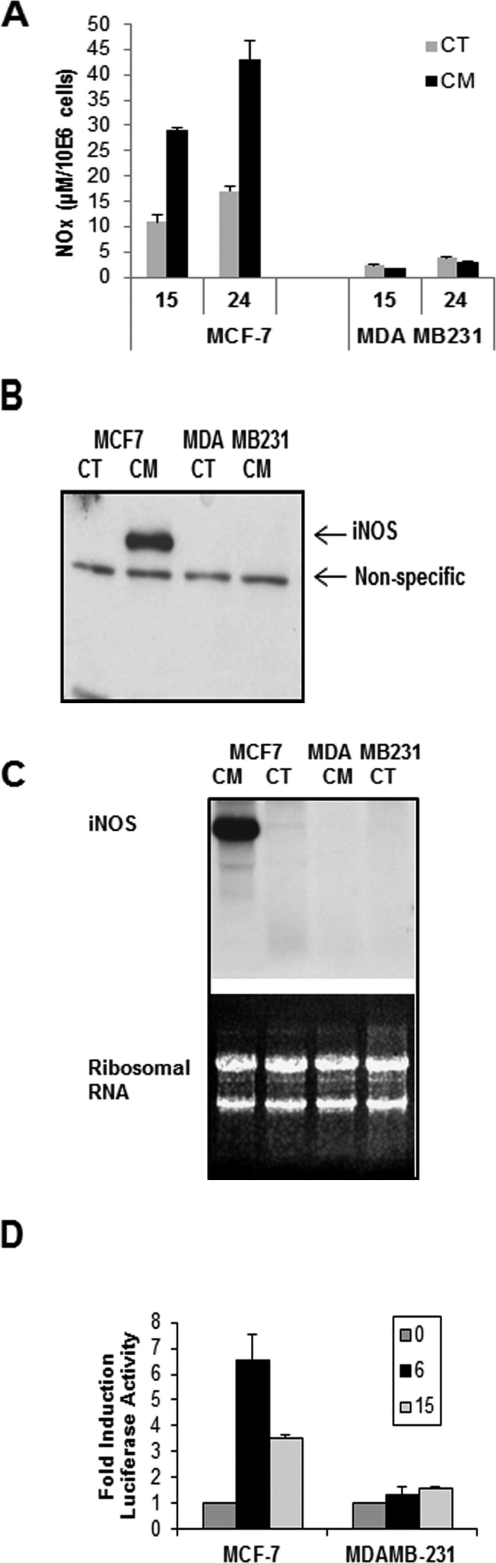
iNOS expression in MCF-7 and MDA-MB-231 cells. (**A**) NOx (nitrites and nitrates) accumulation in the culture medium of control (CT) or cells stimulated with a cytokine mix (CM: Il1-β, IFN-γ and TNF-α) (mean + SD). (**B**) Western blot of control (CT) and stimulated (CM) MCF-7 and MDA-MB-231 cells with a specific antibody to human iNOS. (**C**) Northern blot showing iNOS mRNA accumulation in control (CT) and stimulated (CM) cells of both cell lines. Ribosomal RNA is shown as a loading control. (**D**) Activity of a luciferase reporter gene containing 7 kb of the iNOS promoter after stimulation with cytokines for the specified time. Mean + SD.

While studying the transcriptional regulation of iNOS in breast cancer cell lines (13), we noticed that in general lines such as MCF-7 express the enzyme in response to stimulation with a cytokine mix (Il1-β, IFN-γ and TNF-α) while others such as MDA-MB-231 do not (Figure 1). Assessing iNOS activity by measuring the nitrite and nitrate (NOx) accumulation in the culture media in response to cytokine activation (Figure 1A) shows iNOS activity in MCF-7 cells after 24 h of cytokine stimulation while no NOx can be detected in MDA-MB-231 media. In order to determine the level at which iNOS synthesis is blocked in the MDA-MB-231 cell line, we first examined protein synthesis by western blot that revealed iNOS protein in stimulated MCF-7 but not in MDA-M-231 cells (Figure 1B). Correspondingly, iNOS mRNA is also detected in stimulated MCF-7 but not in MDA-MB-231 (Figure 1C). Consistent with a transcriptional block of iNOS expression, a luciferase reporter construct containing 7 kb of the iNOS promoter displays no activity in response to cytokine stimulation in MDA-MB-231 while MCF-7 cells respond with a seven-fold activation (Figure 1D). As this is an exogenous DNA construct, it is not subject to the epigenetic makeup that governs the cell genome, suggesting that the repression might be driven by transcriptional effectors.

### Transcription factors involved in the differential expression of iNOS

**Figure 2.**
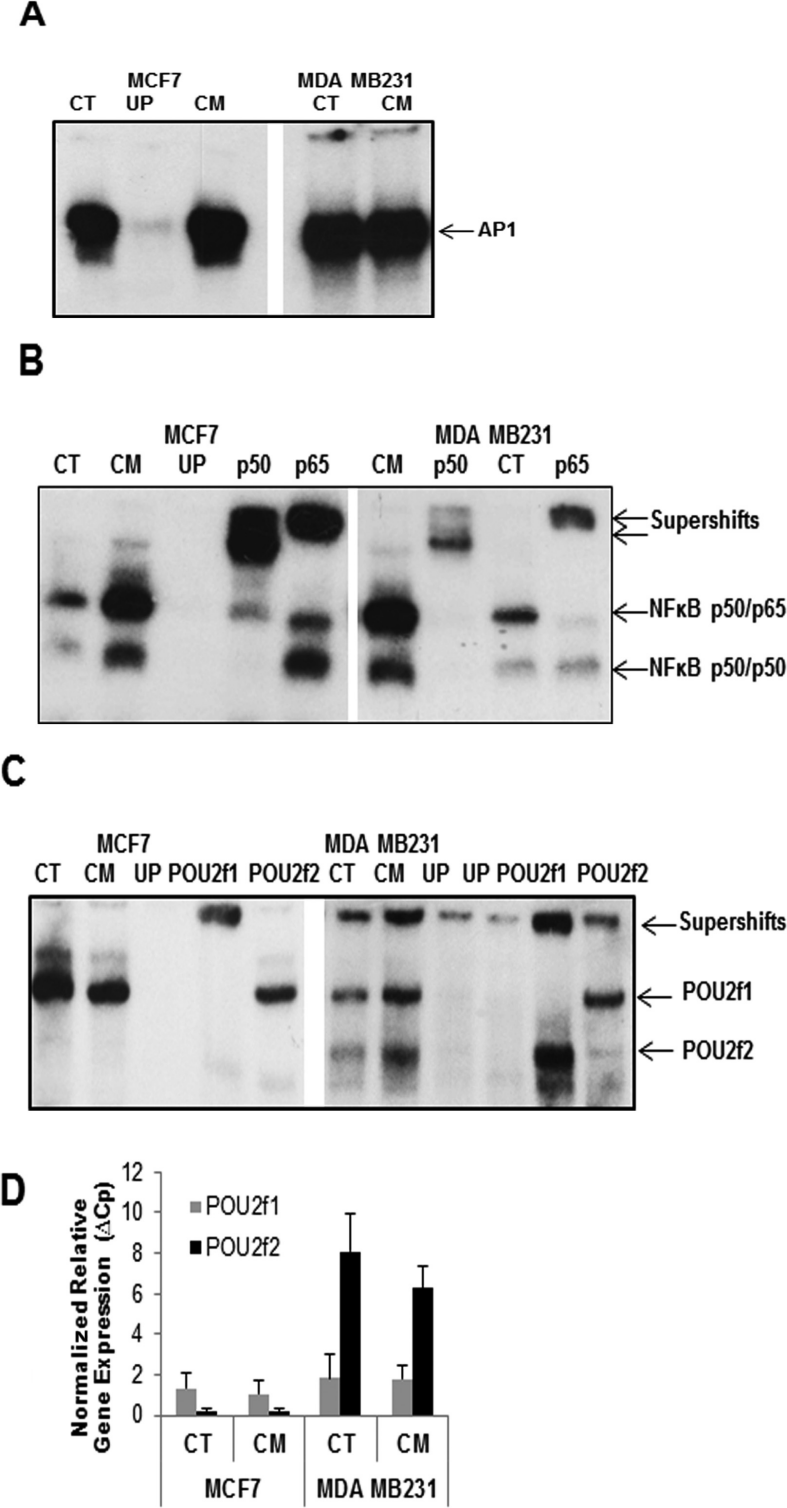
EMSA analysis of transcription factors involved in iNOS transcription. (**A**) AP-1 complex in control (CT) and induced (CM) MCF-7 and MDA-MB-231 cells (2 μg of nuclear extracts). UP: competition with un-labelled probe (**B**) NFκB in nuclear extracts (2 μg) of control (CT) and stimulated (CM) MCF-7 and MDA-MB-231 cells. The components P50 and P65 are demonstrated with specific antibodies. UP: competition with unlabelled probe. (**C**) Octamer factors in nuclear extracts (2 μg) from MCF-7 and MDA-MB-231 cells, control (CT) or induced with cytokines (CM). Specificity of binding is probed with competition with unlabelled probe (UP) and the presence of Oct1 (POU2f1) or Oct2 (POU2f2) is demonstrated with specific antibodies. (**D**) Levels of expression of Oct-1 and Oct-2 in both MCF-7 and MDA-MB-231 cells control (CT) or stimulated with cytokines (CM). A real time PCR was performed using 1 μl cDNA per sample.

Since the blockage of iNOS expression in MDA MB231 cells is transcriptional, we examined some of the key transcription factors induced in both cell lines in response to cytokine stimulation by electrophoretic mobility shift assays (EMSAs) using consensus oligonucleotides. The essential transcription factors implicated in iNOS regulation are Oct-1, NFκB and AP-1 (7,15,16). We found that AP-1 is similarly expressed in both cell lines with and without cytokine stimulation (Figure 2A). NFκB is translocated to the nucleus in both cell lines in response to CM and the DNA-binding complexes consist of the p65-p50 dimers in both cases (Figure 2B). Examination of the Oct factors however showed Oct-1 in MCF-7 while MDA-MB-231 cells show a more complex pattern where Oct-1 as well as Oct-2 were identified with specific antibodies (Figure 2C). The level of the Oct transcription factors in the cell lines was quantified by q-RT-PCR, which revealed that while expression of Oct-1 is similar in MCF-7 and MDA-MB-231 cells with or without cytokine stimulation, the level of Oct-2 is much higher (four-fold) than that of Oct-1 in the MDA-MB-231 cell line (Figure 2D).

**Figure 3.**
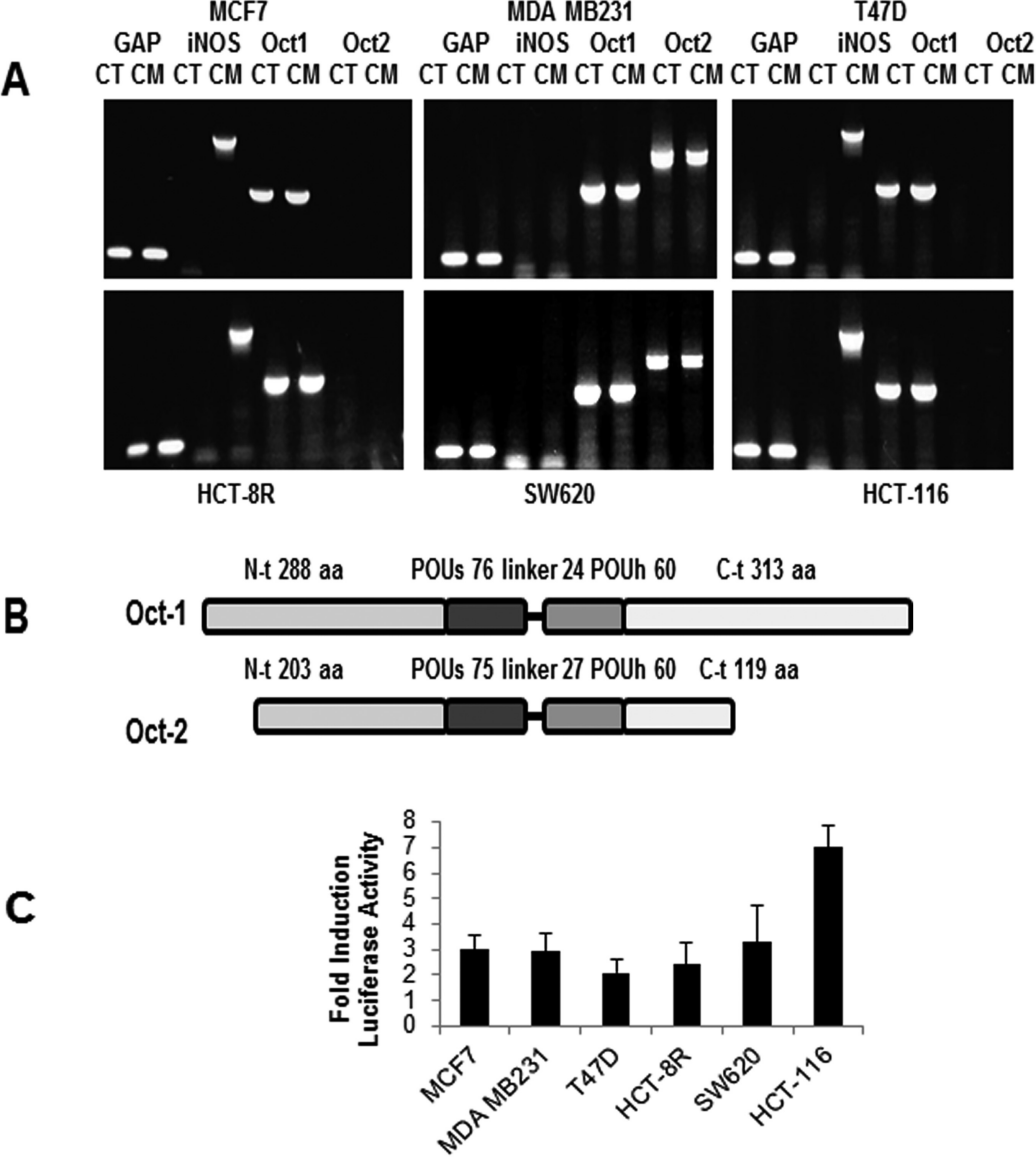
Correlation of Oct-1 and Oct-2 with iNOS expression. (**A**) RT-PCR of control (CT) and cytokine stimulated (CM) cell lines (MCF-7 MDA-MB-231, T47D, HCT-8R, SW620 and HCT-116), showing expression of iNOS, Oct-1 and Oct-2 with GAP as internal control. (**B**) Diagram of the structural differences between Oct-1 and Oct-2. (**C**) Activity of a luciferase reporter gene containing 4 NFκB DNA binding elements in all cell lines after 6h stimulation with agonist TNF-α. Mean + SD.

In order to assess a possible correlation between the presence of Oct-2 and a transcriptional repression of iNOS, expression of these proteins was examined by RT-PCR in various cell lines. Figure 3A shows that all cell lines tested express Oct-1 constitutively, however expression of iNOS in response to cytokine stimulation is only observed in cell lines where Oct-2 is absent. Thus the breast cancer cell line MDA-MB-231 and the colon cancer cell line SW620 show the presence of Oct-2 and no inducibility of iNOS in contrast to MCF-7 and T47D in the former group as well as HCT-8R and HCT-116 in the latter. The structural differences between Oct-1 and Oct-2 are shown diagrammatically in Figure 3B.

As NFκB is essential for iNOS expression, we use a NFκB-luciferase reporter gene construct to assess its functionality and rule out the possibility that iNOs repression in these cell lines is due to a deficiency in this factor. Stimulation with the agonist TNF-α activates NFκB-driven transcription in all cell lines to similar levels, confirming that NFκB is transcriptionally competent in all of them (Figure 3C).

Several isoforms of Oct-2 are reported in the data base and some of them lack important functional domains, so we deemed it crucial to identify the specific isoform present in these cell lines. In order to do this, the FL Oct-2 was amplified by RT-PCR from MDA-MB-231 and SW620 and sequenced. The RT-PCR showed only one band in both cell lines and the sequence obtained identified isoform 1 of Oct-2 (Acc NM_001207025.2, not shown).

### Oct-2 is responsible for the repression of iNOS in cancer cell lines

**Figure 4.**
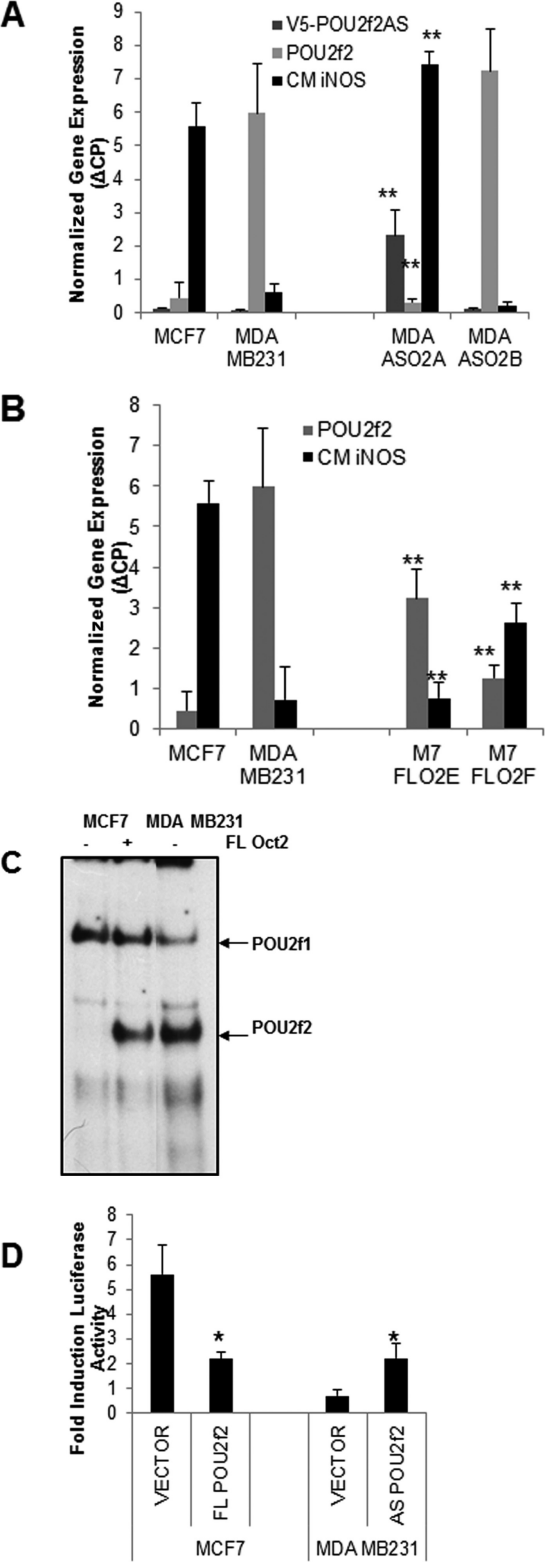
Role of Oct-2 in the repression of iNOS transcription. (**A**) MDA-MB-231 stable transfectant colonies of anti-sense Oct2 were examined by qRT-PCR for expression of Oct-2 (POU2f2) and presence of the vector (V5-POU2f2AS). Transcription of iNOS was estimated with qRT-PCR of exon 12 in both cell lines and selected colonies in cytokine-stimulated (CM) cells. Results are presented as mean + SD **P < 0.001. (**B**) Stable colonies of MCF-7 transfected with full length (FL) Oct-2. Expression of Oct-2 (POU2f2) was assessed by qRT-PCR of the colonies as compared with both parental lines. Transcription of iNOS was estimated with qRT-PCR of exon 12 in both cell lines and selected colonies in cytokine-stimulated (CM) cells. (**C**) EMSA with nuclear extracts (2 ugr) from MDA-MB-231cells and MCF-7 cells transfected (+) or not (−) with a FL Oct2 expression vector using a consensus octamer probe. (**D**) iNOS promoter activation was estimated with the luciferase reporter construct co-transfected with FL Oct-2 into MCF-7 cells and anti-sense Oct-2 in MDA-MB-231 cells stimulated with cytokines for 6 h. Data represents mean and SD, *P < 0.005.

The role of Oct-2 in the repression of iNOS was assessed directly with the design of an anti-sense strategy to enable us to make stable lines. We cloned a 163 bp N-terminal fragment of Oct-2 into a topo TA V5/His expression vector in antisense orientation, transfected it into MDA-MB-231 cells and selected with G418. The colonies obtained were tested for stable expression of the construct using a primer specific for V5. Figure 4A shows that high expression of the anti-sense construct in the selected colonies is accompanied by a strong decrease in Oct-2 mRNA levels. The colonies where a downregulation of Oct-2 was achieved, show restored iNOS transcriptional activation in response to cytokine stimulation that is comparable to that observed in MCF-7 cells (Figure 4A). The reciprocal experiment was performed by transfecting a FL Oct-2 expression vector into MCF-7 cells and generating stable Oct-2-expressing MCF-7 clones. Expression of Oct-2 was confirmed by qRT-PCR (Figure 4B) and the functionality of the introduced gene was assessed by EMSA. Nuclear extracts from the transfected cells showed that expressed Oct-2 is capable of binding the octamer consensus oligonucleotide (Figure 4C). In the successfully transfected MCF-7 colonies, iNOS transcription is greatly reduced as shown in Figure 4B.

The effect of Oct-2 on iNOS transcription was confirmed at the promoter level co-transfecting the iNOS promoter-luciferase reporter construct with either FL or anti-sense (AS) Oct-2 expression vectors (Figure 4D). Expression of FL Oct-2 results in a strong inhibition of promoter activity in response to cytokines demonstrating that Oct-2 directly inhibits iNOS promoter activity. On the other hand the knockdown of Oct-2 significantly reduces transcriptional activity (*P* < 0.005). The lower activity of the reporter gene compared to the levels of luciferase in stimulated MCF-7 cells (Figure 1D), might be due to a less efficient knockdown of Oct-2 in transient transfections and might also be greatly affected by the efficiency of co-transfection.

### Oct-2 binds to the NOSII promoter and prevents PolII recruitment

**Figure 5.**
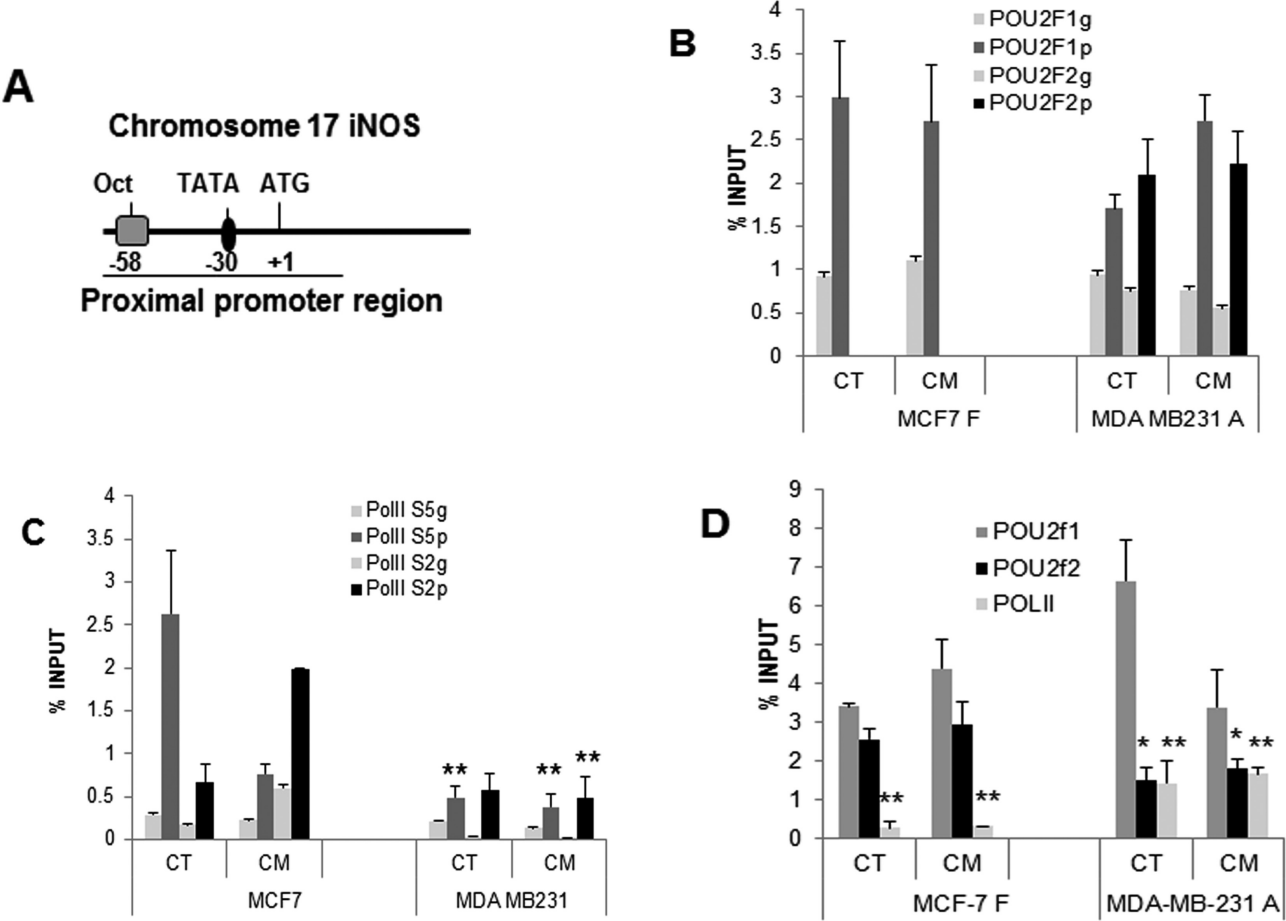
Oct-1 and Oct-2 binding on the iNOS promoter and effect on PolII recruitment. (**A**) Schematic representation of the iNOS proximal promoter where protein binding is detected. (**B**) ChIP with specific antibodies to Oct-1 and Oct-2 followed by a qRT-PCR of the iNOS proximal promoter region that harbours the Oct site (POU2f1p, POU2f2p) or a downstream sequence within the gene (POU2f1g, POU2f2g). MCF-7 and MDA-MB-231 cells were untreated (CT) or stimulated with cytokines (CM) for 2 h prior to immunoprecipitation. Mean + SD. (**C**) ChIP with specific antibodies to RNA PolII phosphorylated on serine 5 (PolII S5) and serine 2 (PolII S2) followed by a qRT-PCR of the iNOS TSS region (PolII S5p, PolII S2p) and a downstream sequence within the gene (PolII S5g, PolII S2g). Data represents mean + SD **P < 0.001. (**D**) Binding of Oct-1 (POU2f1), Oct-2 (POU2f2) and PolII on the proximal iNOS promoter in the Oct-2-expressing MCF-7 and Oct-2 anti-sense MDA-MB-231 cell lines. Data represents mean and SD, *P < 0.05; **P < 0.001.

To unravel the mechanism by which Oct-2 suppresses iNOS expression, we aimed at defining the site of Oct-2 binding within the iNOS promoter. In our previous work we searched the proximal 7 kb of the human iNOS promoter for octamer binding sites and we showed that only the proximal site located close to the TATA box binds Oct-1 (7). Therefore we examined the binding of Oct transcription factors to the proximal promoter of iNOS (Figure 5A) *in vivo* using ChIP. As shown in Figure 5B, we found Oct-1 on the proximal promoter in both cell lines however in MDA-MB-231 cells binding of both Oct-1 and Oct-2 is detected. The specificity of the chromatin binding was verified by testing a sequence downstream within the gene where binding is not expected (Figure 5B light bars labelled g).

The effect of the POU factors on PolII binding and activation was analysed by ChIP using specific antibodies to phosphorylated forms of PolII namely, serine-5 phosphorylation (PolII S5) which corresponds to the PolII engaged in initiation of transcription and serine-2 phosphorylation (PolII S2) that is the elongating enzyme. The results show that in resting MCF-7 cells the predominant binding corresponds to PolII S5 indicating initiation of transcription while binding of the elongation-active PolII S2 is low. Once stimulated, PolII S2 becomes predominant while PolII S5 decreases (Figure 5C). In contrast, the overall levels of PolII present on the iNOS promoter in MDA-MB-231 cells are very low independently of stimulation. The specificity of these reactions was verified as above (Figure 5C light grey bars labelled g).

The correlation between the binding of the two POU factors and recruitment of PolII was confirmed with the stably transfected clones. In this case, specificity of the reactions was also verified as above (not shown). Figure 5D shows that when Oct-2 is introduced into MCF-7 cells, PolII is no longer recruited to the promoter, whereas the knock down of Oct-2 in MDA-MB-231 cells restores PolII binding.

### Oct-1 and Oct-2 form a higher order complex on the iNOS promoter that controls transcription

**Figure 6.**
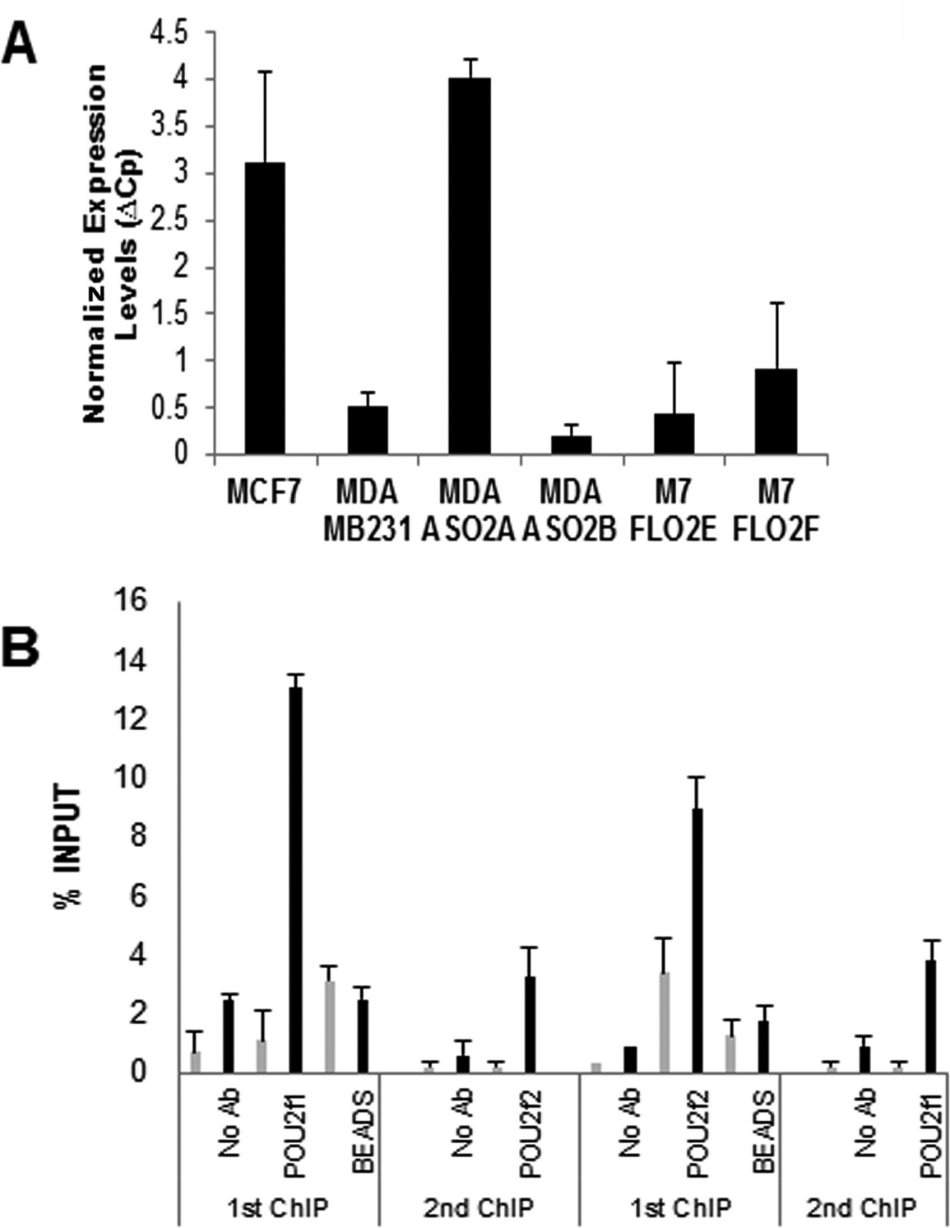
PolII function and POU complex formation on the iNOS promoter. (**A**) Transcription of iNOS exon 1 as estimated by qRT-PCR for the parental (MCF-7 and MDA-MB-231) and modified cell lines: MDA-MB-231anti-sense Oct-2 (MDA ASO2A, MDA ASO2B) and MCF-7 FL Oct-2 (M7 FLO2E, M7 FLO2F). Mean + SD. (**B**) Sequential ChIP. MDA-MB-231 cells were crosslinked and chromatin was precipitated with no antibody (No Ab) or with either Oct-1 (POU2f1) or Oct-2 (POU2f2) antibodies (first ChIP). The protein–protein interactions were disrupted, the stripped beads processed to check efficiency (BEADS) and the released chromatin was re-precipitated with the reciprocal antibody Oct-2, Oct-1 or no antibody. After reverse crosslinking precipitated iNOS proximal promoter was quantified by qPCR (black bars) and a downstream region within the gene was used as specificity control (light grey bars). Data represents mean and SD.

Consistent with the binding of PolII S5, transcription of iNOS is initiated in resting MCF-7 cells as judged by the transcription of exon1 (Figure 6A). No exon1 is transcribed in MDA-MB-231 cells under these conditions. Furthermore, when we analyse the stable clones described above, exon1 transcription is observed in MDA-MB-231 cells where expression of Oct-2 has been knocked-down, while MCF-7 clones expressing Oct-2 lose exon1 transcription (Figure 6A).

Taken together, our observations of total iNOS repression in the presence of Oct-2 and the equivalent binding on the iNOS promoter of both Oct-1 and Oct-2, suggests the formation of a higher order complex of these factors on the promoter. We corroborated this hypothesis by performing a sequential ChIP where the precipitated chromatin with either Oct-1 or Oct-2 was re-precipitated with the reciprocal factor (Figure 6B). The specificity of the binding detection was verified using primers within the gene targeting a non-related region (Figure 6B light grey bars). The small recovery of material after the second IP is due to the extensive washing and some loss of material with the beads from the first IP. The results show that both Oct-1 and Oct-2 bind to the iNOS proximal promoter simultaneously, forming a higher order complex and that this interaction has functional consequences for transcription of iNOS.

## DISCUSSION

The Oct transcription factors coordinate a variety of cell responses to internal cues such as pluripotency and differentiation as well as extracellular signals determining proliferation, survival and death. The function of the Oct factors is largely determined by the timing and cell specificity of their expression. Though Oct-2 is thought to be restricted to B lymphocytes and neuronal cells, it has also been detected in various non-B cancer cell lines (17,18). The former study detected Oct-2 specifically in the SW480 and H1299 cancer cell lines, but not in others such as HT29. Interestingly, SW480 is derived from the same patient as the SW620 line we examine here and just as SW620 and MDA-MB-231, does not express iNOS ((19), Pance *et al* unpublished data). In contrast, HT29 responds to cytokine stimulation with a strong induction of the enzyme ((20), unpublished data). Similar to our findings regarding iNOS expression, immunoglobulin gene (Ig) transcription was found in some non-B cancer cell lines including HT29 and a proximal octamer element bound by Oct-1 was essential for this activity. Crucially, the panel of lines examined revealed that in those cancer cell lines expressing Oct-2 (SW480 and H1299), no Ig promoter activity could be detected (17).

Consistent with an equivalent DNA binding domain, both Oct-1 and Oct-2 are detected on the proximal oct site of the iNOS promoter in the MDA-MB-231 cell line which cannot induce iNOS. Our EMSA with the Oct consensus sequence shows that the antibodies for Oct-1 and Oct-2 used in this study are specific and therefore the results from the ChIP experiments indicate binding of both Oct-1 and Oct-2 to the same element in the iNOS promoter. Furthermore the binding of both factors in equivalent proportion together with the total repression of iNOS in the Oct-2-expressing cells suggests the formation of a higher order complex on the promoter, which is confirmed by sequential ChIP experiments. Indeed associations of oct proteins with themselves as well as other transcription factors are well documented (21–23). Complex formation involving Oct factors depends on an extended DNA sequence that can accommodate dimers and in some cases select for specific partners (21,24). A thorough study of the DNA binding properties of these factors showed that the octamer sequence alone only allows monomeric binding of Oct-1 and Oct-2, while longer DNA sequences, such as the native promoter, support the formation of homo- and hetero-dimers of both factors (25). This report confirms our finding that using the Oct consensus sequence alone in EMSA favours monomeric binding of the Oct factors while native chromatin is prone to higher order complex formation as detected by our ChIP experiments.

In our previous work, we showed that Oct-1 binding to the iNOS proximal promoter results in PolII recruitment and transcription initiation that is paused at exon1 (7). However when Oct-2 is bound to the promoter, PolII is not recruited and as a consequence transcription does not proceed, pointing at a mechanism of the differential transcriptional activity between these two factors. Even though Oct-1 and Oct-2 have high homology of the POU DNA-binding domains (87% excluding the linker region) that allows them to bind the same DNA sequences, there are functional differences between them. For example Oct-1 but not Oct-2 can recruit the herpes virus trans-activator VP16 and thereby acquire the capacity to stimulate transcription from particular sites (26)erHerr. In contrast to the POU domain, the N- and C- terminal activation domains of Oct-1 and Oct-2 bear little homology (see Figure 3B). The N-terminal domain differs in size and has a 28.5% homology but both have a high content of glutamines. The C-terminal domain of Oct-1 is 194aa longer than that of Oct-2 with a 12% homology, but there are also similar features consisting of stretches rich in prolines, serines and threonines (27). Some of the most striking functional differences observed between these factors are associated to their distinct activation domains. The ability of Oct-2 to transactivate the Ig genes in B lymphocytes is not shared with Oct-1 and this activity was mapped to the C-terminal region of Oct-2 (28). Some of these functional differences have been attributed to post-translational modifications of the two factors. Thus Oct-2 but not Oct-1 has the capacity to activate the B-globin promoter in non-immune cells and this differential regulation depends on the specific phosphorylation of Oct-2 C-terminal domain (3). Oct-1 on the other hand has been reported to be heavily phosphorylated, mainly in the N-terminal glutamine-rich domain. The DNA-dependent protein kinase (DNA-PK) is responsible for this modification in response to DNA damage, which leads to a decrease in Oct-1 transactivation capacity manifested by a strong decrease in the expression of target genes such as H2B. Though phosphorylation does not modify Oct-1 binding to the DNA, it dramatically reduces the binding of PolII and TATA-binding protein (TBP) to the promoter (4). These observations are consistent with our results and support our proposal that an important part of Oct-1 transcriptional activity is the recruitment of PolII to the target promoter. Other post-translational modifications of Oct-1 activation domains, such as O-GlcNAc and ubiquitination have also been reported that modulate transcriptional activity (29).

Another property that distinguishes the Oct factors might rely on how they affect the surrounding chromatin of their binding sites. Oct-1 has been reported to have chromatin remodelling activity through recruitment of the histone demethylase Jmjd1 or deacetilating complex NuRD/Mi-2. This is important for specialized regulation such as maintaining Jmjd1a at the IL-2 promoter in resting pre-stimulated T cells to remove H3K9me2 marks and protect DNA from methylation to promote a stronger expression associated with secondary stimulation (5). The more open chromatin favoured by Oct-1 might facilitate assembly of the PIC and PolII recruitment.

The interplay of the POU factors in the different cell types where they are expressed leads to a fine tuned regulation of protein expression which is due to differential binding, as well as cooperative activity between the various members of this family as well as components of the transcriptional machinery. This capacity allows a differential regulation of the expression of immunoglobulins and IL-2 in lymphocytes for example while at the same time perhaps making sure that proteins with potentially harmful effects such as iNOS are repressed in particularly sensitive cell types. At the same time, it is possible that some cancer cell lines have overexpressed Oct-2 as a mechanism to regulate the proteome towards cell proliferation and survival that might play a role in the development of tumorigenesis. Furthermore, the susceptibility to post-translational modifications that modulate POU factors activity confers the ability to orchestrate the cell response to extracellular signals and stress cues.
